# Supplementary material for: Short-Term Exposure to Bisphenol A Does Not Impact Gonadal Cell Steroidogenesis In Vitro
Source: Cells. 2023 Jun 2;12(11):1537. doi: 10.3390/cells12111537 (PMC10252311; doi:10.3390/cells12111537)
Supplement: Supplementary file 1 [file cells-12-01537-s001.zip › Supplementary Figures S1 and S2.pptx]

## Slide 1
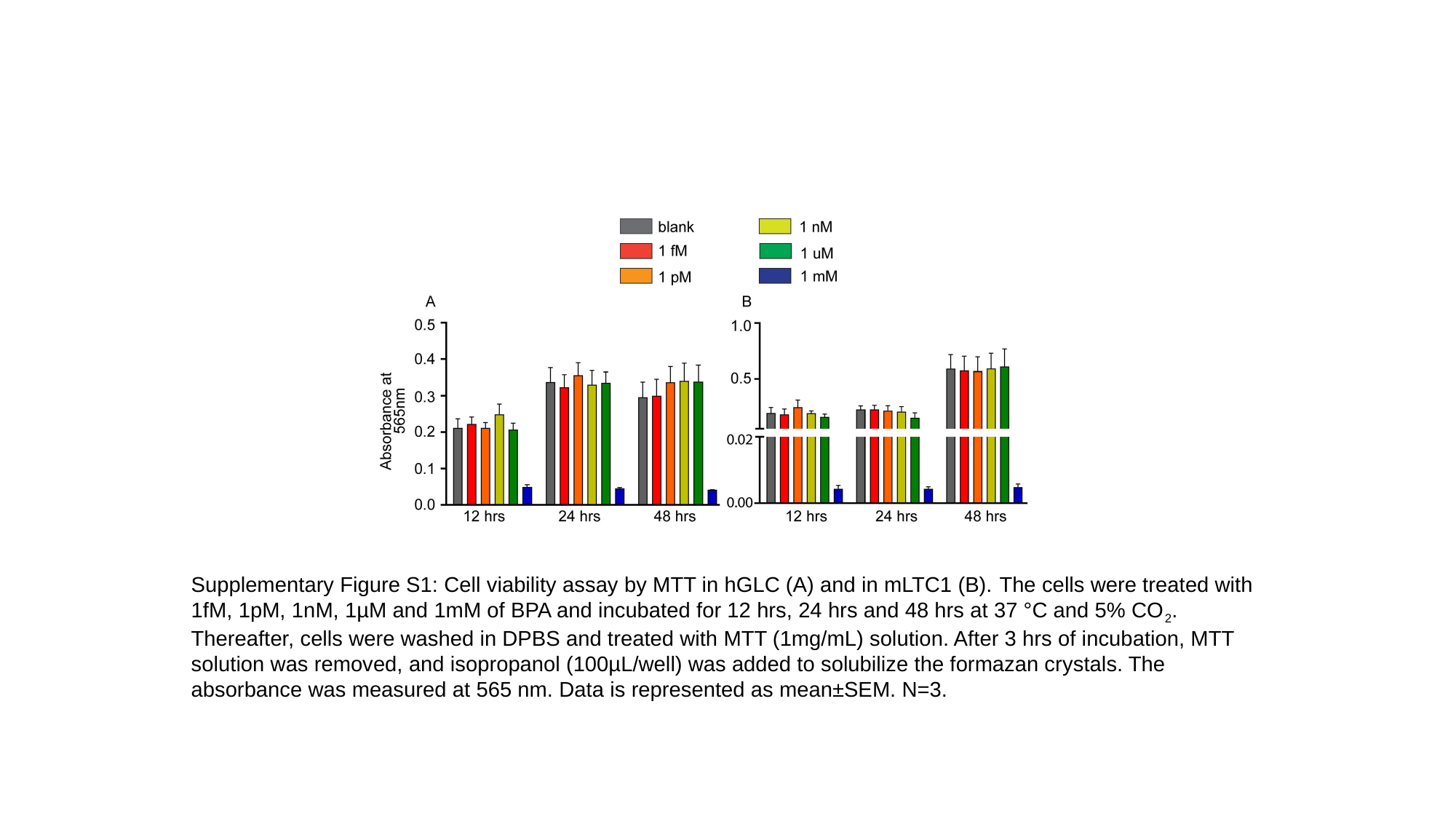

Supplementary Figure S1: Cell viability assay by MTT in hGLC (A) and in mLTC1 (B). The cells were treated with 1fM, 1pM, 1nM, 1µM and 1mM of BPA and incubated for 12 hrs, 24 hrs and 48 hrs at 37 °C and 5% CO2. Thereafter, cells were washed in DPBS and treated with MTT (1mg/mL) solution. After 3 hrs of incubation, MTT solution was removed, and isopropanol (100µL/well) was added to solubilize the formazan crystals. The absorbance was measured at 565 nm. Data is represented as mean±SEM. N=3.

## Slide 2
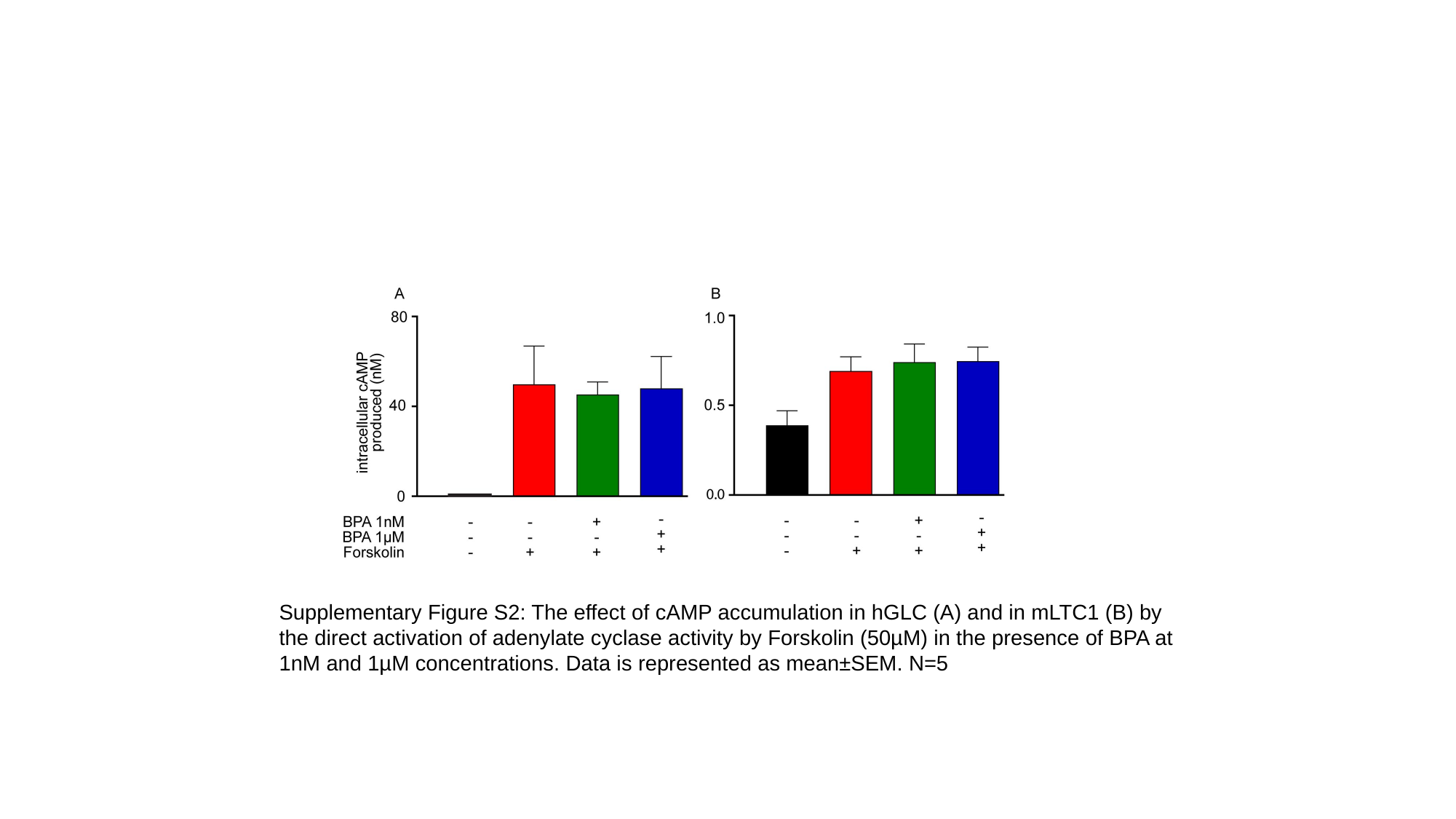

Supplementary Figure S2: The effect of cAMP accumulation in hGLC (A) and in mLTC1 (B) by the direct activation of adenylate cyclase activity by Forskolin (50µM) in the presence of BPA at 1nM and 1µM concentrations. Data is represented as mean±SEM. N=5
